# Transient abnormal myelopoiesis in a premature infant with Down syndrome: A case report

**DOI:** 10.1097/MD.0000000000043178

**Published:** 2025-07-18

**Authors:** Jin Wang, Dan Wang, Xuwei Tao, Tingting Li, Lingkong Zeng, Shi Wang

**Affiliations:** a Department of Neonatology, Wuhan Children’s Hospital (Wuhan Maternal and Child Healthcare Hospital), Tongji Medical College, Huazhong University of Science & Technology, Wuhan, Hubei Province, China.

**Keywords:** case report, premature infant, transient abnormal myelopoiesis

## Abstract

**Rationale:**

Transient abnormal myelopoiesis with mutations in *GATA1* gene can be self-alleviated after 3 to 4 months of birth in term infant, however, the premature infant with this disease in our research achieved remission earlier.

**Patient concerns:**

A 10-hours-old girl was diagnosed with transient abnormal myelopoiesis with *GATA1* mutation.

**Diagnosis:**

Transient abnormal myelopoiesis in a premature infant was suspected.

**Interventions:**

The patient received anti-infection, liver protection, hydration, and alkalization treatments for leukocytosis.

**Outcomes:**

After admission, the infant was diagnosed with TAM with *GATA1* mutation after completing bone marrow cytology, whole-exon gene detection, and FISH detection. The *GATA1* gene mutation of this baby turns negative a month later.

**Lessons:**

Transient abnormal myelopoiesis differs from congenital leukemia. Most children can self-alleviate after 3 to 4 months of birth, and *GATA1* mutation turns negative. Since some children with transient abnormal myelopoiesis may develop myeloid leukemia of Down syndrome, continuous follow-up is required once transient abnormal myelopoiesis is diagnosed for early detection and treatment.

## 1. Introduction

Transient abnormal myelopoiesis (TAM) and myeloid leukemia of Down syndrome (ML-DS) are associated with myeloid proliferation in trisomy 21 patients.^[1]^ Unlike other types of congenital leukemia and myeloproliferative tumors in children, both are present in children with Down syndrome with mutations in *GATA1*.^[2]^ The encoding product of the *GATA1* gene plays an important role in the differentiation of bone marrow primitive red blood cells and megakaryocytes.^[1,2]^

TAM is self-limited in that it resolves 3 to 4 months after birth in more than 80% of children with TAM or recessive TAM and the child becomes negative for *GATA1* mutations.^[1,3]^ Approximately 10% of patients die, and the risk factors associated with early death after birth include hyperleukocytosis, prematurity, and elevated bilirubin levels.^[3–5]^

There is no effective treatment for TAM, and the purpose of current treatment approaches, including low-dose cytarabine (LDAC), exchange blood transfusion (ET) therapy, and systemic steroid therapy, is to relieve clinical symptoms.^[6,7]^ However, all treatment options carry significant risks for newborns; therefore, the treatment method is usually selected based on the presence of life-threatening conditions.^[7,8]^

To help clinicians better understand the characteristics of TAM progression in infants, we introduce a case of TAM in a premature infant and the changes in the sites of mutation on the GATA1 gene during the follow-up process.

## 2. Case presentation

A 10-hour-old girl, with a birth weight of 2000 g, was born at 33^+3^ weeks of gestation by cesarean section in December 2022; her mother was healthy during pregnancy. After birth, she was admitted to the NICU of our hospital because of a poor response. After admission, the newborn was found to have a wide forehead and widened eye spacing. The laboratory tests revealed a significantly elevated white blood cell count. The child’s parents were informed of the study and provided informed consent. This study was approved by the Medical Ethics Committee of the hospital (approval number: 2021R204-E03), and our research obtained the written informed consent of the legal guardians of her patients.

Relevant examinations were completed after admission, and the results of blood analyses within 24 hours after birth and at 1 day, 3 days, 1 month, and 3 months after birth are shown in Table 1. The proportions of original cells examined via blood analysis within 24 hours, 1 day, and 3 days after birth were 60%, 65%, and 58%, respectively. Liver function tests revealed that the alanine aminotransferase level was 110 U/L (8–71 U/L), aspartate aminotransferase level was 103 U/L (35–140 U/L), and gamma-glutamyltranspeptidyase level was 403 U/L (9–150 U/L). The child’s renal function tests and blood glucose and electrolyte levels were normal. Urine analysis and routine stool examination revealed no abnormalities. C-reactive protein (CRP) and procalcitonin (PCT) levels were within normal ranges. No pathogenic bacteria were detected in whole blood bacterial cultures.

**Table 1:** Results of blood analysis during the course of the disease.

Time (d)	White blood cell (5.0–20.0*10^9^)	Neutrophils (2.0–10.0*10^9^)	Lymphocytes (2.0–10.0*10^9^)	Monocytes (2.0–10.0*10^9^)	Hemoglobin (g/L)		Red blood cell (4.1–5.74*10^12^)	Platelet (144–450*10^9^)
0	105.00	35.70	54.50	14.51	119	3.29		1181
1	73.12	22.45	25.74	24.06	126	3.69		1223
3	67.68	16.11	19.63	30.46	112	3.20		1354
30	21.24	8.45	4.96	5.87	103	2.97		851
90	19.36	9.12	6.24	3.52	98	2.65		798

**Figure 1.**
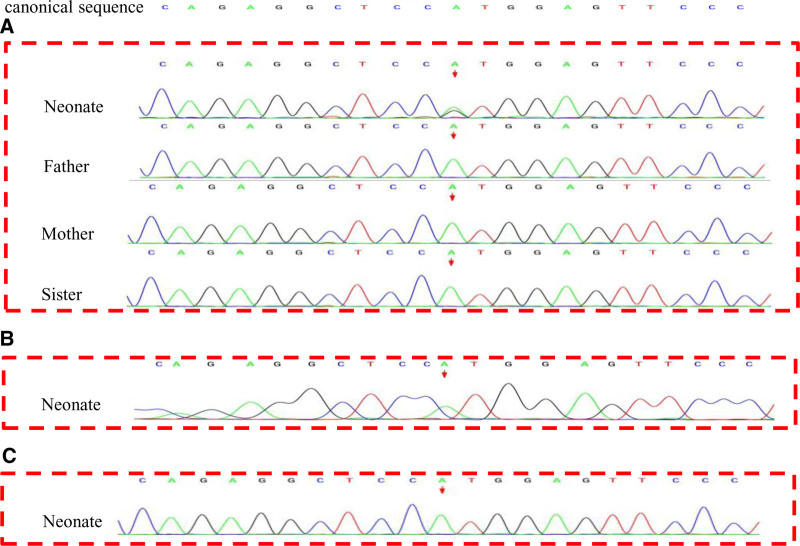
Sanger sequencing results of *GATA1* gene in this newborn and her family members. The result showed that the child had a new mutation of *GATA1* gene c.1(exon 2) A>G (p. Met1Ala) which was a heterozygous mutation, both her parents and sister were wild-type, the red arrow indicated the location of the gene mutation (A). Sequencing results of *GATA1* gene indicated no mutation at 1 and 3 mo (B and C).

FISH of the child’s peripheral blood was positive for chromosome 21, and whole-exon gene detection was performed with the consent of the guardian. The results revealed that the newborn had a new heterozygous mutation of *GATA1*, c.1 (exon 2) A>G (p. Met1Ala) (Fig. 1A). Both her parents and sister were wild-type, and the protein was predicted to be harmful. In accordance with variation standards and guidelines proposed by the American College of Medical Genetics and Genomics (ACMG), the variant was determined to be likely pathogenic (PVS1 Moderate + PS3 + PM2 Supporting).

## 3. Treatment

After admission, the patient was administered symptomatic treatment, including anti-infection therapy (ceftazidime), oxygen therapy, reduced glutathione (liver protection), fluid therapy (hydration), and alkalization therapy. The patient’s clinical symptoms improved, her spontaneous breathing was stable, and the volume of milk consumed during oral feeding gradually increased. The patient was discharged 12 days after admission. The patient was followed up at 1 and 3 months after discharge as an outpatient, and Sanger sequencing of the *GATA1* gene was reviewed. No mutations were found in the test results (Fig. 1B and C), and outpatient follow-up is still ongoing.

## 4. Discussion

In contrast to other types of congenital acute leukemia and myeloproliferative tumors, TAM and ML-DS occur in children with trisomy 21 syndrome and are often accompanied by mutations in the key hematopoietic transcription factor *GATA1* gene. Therefore, these diseases have unique clinical and biological characteristics.^[3,4]^ Clinical analysis of data from ML-DS children revealed that most ML-DS children developed TAM^[5,6]^ before the age of 5. At present, TAM is diagnosed on the basis of a proportion of peripheral blood original cells >10% and *GATA1* gene variation; however, invisible TAM is diagnosed on the basis of a proportion of peripheral blood original cells < 10% and *GATA1* gene variation.^[7]^ The proportion of peripheral blood primitive cells was >10% before and after admission, and FISH of the patient’s peripheral blood was positive for chromosome 21. The findings mentioned above and the results of the whole-exon gene examination were consistent with the TAM diagnosis.

The development process of TAM in ML-DS patients is as follows: first, the normal embryo obtains extra chromosome 21; second, the fetus with trisomy 21 develops mutations in the *GATA1* gene; and finally, the child with trisomy 21 and the GATA1 mutation develop ML-DS^[8–11]^ when they have mutations in other related genes, such as *RAD21*, *EZH2*, *KRAS*, and *CTCF*. In this study, after the diagnosis of TAM, *GATA1* was sequenced at 1 month of age and 3 months of age. The patient was negative for the *GATA1* gene mutation and was still under continuous follow-up. Most newborns are diagnosed with TAM between 3 and 7 days after birth, whereas most children are diagnosed within 2 months after birth. Occult TAM constitutes approximately 10 to 25% of all cases of TAM.^[12]^ Approximately 80% of TAM and invisible TAM cases are self-limited, meaning that they resolve within 3 to 4 after birth and the child becomes negative for the GATA1 mutation. These children did not need treatment. However, in the event of a life-threatening condition, such as obvious edema, liver disease, a significant increase in the white blood cell count, disseminated intravascular coagulation, or even heart failure, chemotherapy is beneficial^[3,12]^ for symptom remission. The remaining 20% of children develop ML-DS, and continuous chemotherapy is needed before some children can be clinically treated.^[3]^

The common clinical symptoms in children with TAM include leukocytosis, thrombocytopenia, liver enlargement, spleen enlargement, jaundice, respiratory distress, pleural effusion, ascites, and bleeding tendency, with thrombocytopenia, liver enlargement, and spleen enlargement being the most common.^[13]^ After admission, the child’s physical examination revealed significant liver enlargement, and the auxiliary examination revealed an elevated transaminase level, indicating liver involvement. At the follow-up, after the remission of TAM, the child’s transaminase level had gradually returned to the normal range, but the amount of decrease in liver size was not obvious, and follow-up is still ongoing.

Leukocytosis is a common clinical manifestation in neonates and can be divided into infectious and noninfectious types. Few children with the noninfectious type develop malignant hyperplasia of the bone marrow. Therefore, identifying malignant proliferative diseases of the bone marrow as early as possible is crucial.^[14,15]^ Since infectious diseases cannot be ruled out before a definitive diagnosis, most of the reported cases were treated with broad-spectrum antibiotics to prevent infections, and anti-infection therapy was paused after evidence supported it.^[15–17]^

There are 3 main types of neonatal leukemia: neonatal acute leukemia, TAM, and juvenile myeloid monocytic leukemia. Diagnosis requires bone marrow cytology, peripheral cytology, and genetic testing, and abnormal peripheral blood findings are the main manifestations in children with TAM.^[17]^ Before admission, the child in this study exhibited signs of peripheral blood leukocytosis, and peripheral blood cytology after transfer to our hospital revealed a significantly increased proportion of original cells, which was consistent with the diagnosis of neonatal leukemia.

However, owing to the child’s clinical presentation and FISH of peripheral blood being positive for chromosome 21, further examination revealed that the child had a *GATA1* gene mutation. At follow-up, the child was negative for a *GATA1* mutation, and the final diagnosis was confirmed as TAM. Most children with TAM have non-life-threatening clinical symptoms; however, a few children with TAM are critically ill after onset and consequently suffer multiple organ damage. Liver failure, which is the most common cause of death in children with TAM, can lead to the progressive worsening of jaundice, coagulation dysfunction, and even disseminated intravascular coagulation.

The limitation of our is the sample size. If we want to further clarify that the remission time of this disease in the premature infant group is shorter, the sample size needs to be further increased. At the same time, due to the insufficient follow-up time, continuous follow-up is required to understand the further changes in the condition of the infant.

In conclusion, the presence of infection, elevated white blood cell counts, and decreased hemoglobin and albumin levels at the time of diagnosis are independent factors contributing to the death of children with TAM.^[17–19]^ Owing to the possibility of ML-DS in children with TAM, continuous follow-up after TAM is diagnosed is crucial for the early detection and treatment of ML-DS.

## Author contributions

**Methodology:** Jin Wang, Dan Wang.

**Supervision:** Xuwei Tao, Tingting Li, Lingkong Zeng.

**Writing – original draft:** Jin Wang, Shi Wang.

**Writing – review & editing:** Shi Wang.
